# Maresin1 Alleviates Metabolic Dysfunction in Septic Mice: A ^1^H NMR-Based Metabolomics Analysis

**DOI:** 10.1155/2019/2309175

**Published:** 2019-01-16

**Authors:** Yu Hao, Hong Zheng, Ruo-Han Wang, Hui Li, Li-Li Yang, Suwas Bhandari, Yong-Jian Liu, Jun Han, Fang Gao Smith, Hong-Chang Gao, Sheng-Wei Jin

**Affiliations:** ^1^Department of Anaesthesia and Critical Care, Second Affiliated Hospital of Wenzhou Medical University, Zhejiang 325027, China; ^2^Institute of Metabonomics & Medical NMR, School of Pharmaceutical Sciences, Wenzhou Medical University, Zhejiang 325035, China; ^3^Academic Department of Anaesthesia, Critical Care, Pain and Resuscitation, Birmingham Heartlands Hospital, Heart of England National Health Service Foundation Trust, Birmingham B9 5SS, UK

## Abstract

Maresin1 (MaR1), a new anti-inflammatory and proresolving lipid mediator, has been proven to exert organ-protective effects in septic animal models. However, the potential mechanisms are still not fully elucidated. In this study, we sought to explore the impact of MaR1 on metabolic dysfunction in cecal ligation and puncture- (CLP-) induced septic mice. We found that MaR1 significantly increased the overall survival rate and attenuated lung and liver injuries in septic mice. In addition, MaR1 markedly reduced the levels of proinflammatory cytokines (TNF-*α* and IL-6) and alleviated mitochondrial damage. Based on a ^1^H NMR-based metabolomics analysis, CLP-induced septic mice had increased levels of acetate, pyruvate, and lactate in serum and decreased levels of alanine, aspartate, glutamate, and fumarate in lungs. However, these metabolic disorders, mainly involving energy and amino acid metabolism, can be recovered by MaR1 treatment. Therefore, our results suggest that the protective effects of MaR1 on sepsis could be related to the recovery of metabolic dysfunction and the alleviation of inflammation and mitochondrial damage.

## 1. Introduction

Sepsis is a life-threatening clinical syndrome characterized by multiple organ dysfunctions due to uncontrolled host inflammatory responses towards infection or injury [1]. Sepsis is becoming a leading cause of morbidity and mortality globally, because to date no efficient antisepsis therapy is available [2]. It is, therefore, important to discover specific and effective drugs to treat sepsis.

Maresin1 (MaR1), derived from docosahexaenoic acid (DHA) by macrophages via 12-lipoxygenase [3], possesses potent anti-inflammatory and proresolving properties in vitro and vivo [4, 5]. A recent research showed that MaR1 mitigated the lipopolysaccharide (LPS) level and enhanced the bacteria clearance in septic mice [6]. Our previous study indicated that MaR1 improved alveolar fluid clearance via upregulation of epithelial sodium channel expression in an LPS-induced acute lung injury model [7]. However, the potential mechanisms of MaR1 for sepsis treatment are still far from being fully understood.

Metabolomics is an “omics” technique that attempts to analyze all low-molecular-weight metabolites in biological samples and explore metabolic mechanisms underlying disease development or drug treatment [8]. Metabolic alterations can be revealed by metabolomic methods for early diagnosis and prognosis in sepsis-induced acute lung injury [9–11]. Moreover, NMR-based metabolomic results showed that the levels of metabolites involved in energy metabolism and inflammatory process were disrupted in the animal model of sepsis [12, 13]. In our previous study, MaR1 significantly decreased the lactate level in CLP-induced septic mice [14]. We also found that MaR1 reversed the mitochondrial dysfunction by upregulating expressions of the mitochondrial respiratory chain enzyme complexes COX I and COX IV in murine sepsis [14], suggesting that MaR1 may influence the metabolic responses, but the metabolic effects of MaR1 in sepsis need to be elucidated. In the present work, we analyzed metabolic profiles of serum, lung, and liver samples in CLP-induced sepsis mice after MaR1 treatment and aimed to explore the MaR1-altered metabolic pathways in sepsis.

## 2. Materials and Methods

### 2.1. Materials

**Figure 1:**
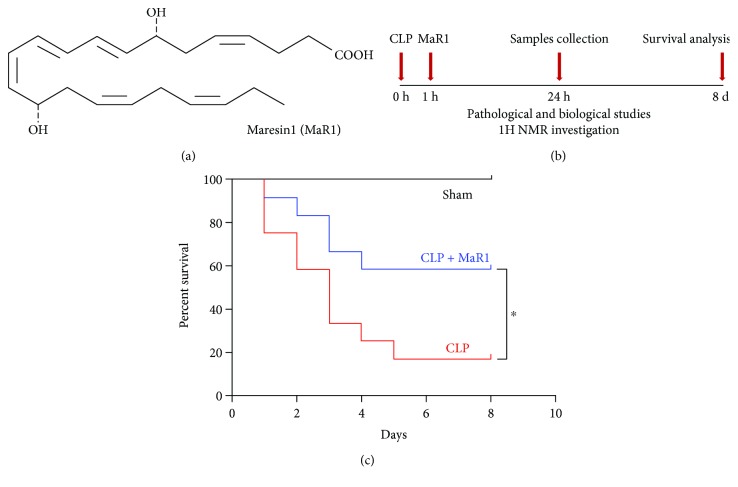
MaR1 improved the survival rate of mice in cecal ligation and puncture- (CLP-) induced septic model (a); the chemical structure of MaR1 (b); experimental schedule (c). MaR1 (100 ng/mice, i.p.) was administered to C57BL/6 mice 1 h after surgery, and an equal volume of saline was given in both the sham and CLP groups. The survival rate was observed for 8 days. Survival of the two subgroups was estimated by Kaplan–Meier survival curves; comparisons were performed by the log-rank test (*n* = 12), ^∗^*P* < 0.05.

Maresin1 (7,14-dihydroxydocosa-4Z,8Z,10,12,16Z,19Z-hexaenoic acid, MaR1) was from Cayman Chemical Company (Ann Arbor, MI, USA). The chemical structure of MaR1 is shown in Figure 1(a). TNF-*α* and IL-6 ELISA kits were from R&D Systems (Minneapolis, MN, USA). Blood biochemistry assay kits (ALT/AST) were from Jiancheng Bioengineering Institute (Nanjing, Jiangsu, China).

### 2.2. Animals, Experimental Procedure, and Treatments

Eight-week-old male C57B6/L mice weighing 20–25 g (Shanghai Experimental Animal Centre, China) were housed four per cage and maintained in a specific pathogen-free room with controlled temperature (22–24°C) and humidity (60–65%) under a 12 h light/dark cycle. The mice were given standard laboratory chow and water ad libitum. Principles of laboratory animal care were followed, and all procedures were conducted according to the guidelines established by the National Institutes of Health (NIH Publication No. 85-23, revised 1996), and every effort was made to minimize suffering. This study was approved by the Animal Care and Use Committee of Wenzhou Medical University.

Mice were anesthetized with 2% sodium pentobarbital (80 mg/kg, intraperitoneally) and randomly assigned to three groups: sham group, CLP group, and CLP + MaR1 group (MaR1: 100 ng/mice, intraperitoneally). Sepsis was induced surgically by CLP as described previously [15]. Midline abdominal incision was made after the abdomen was disinfected and the cecum was exposed. Then, the cecum was ligated below the ileocecal valve and a through-and-through puncture was performed with a 20-gauge needle for mid-grade sepsis. Lastly, it was relocated into the abdominal cavity without spreading faeces from the cecum onto the abdominal wall wound margins. The sham mice underwent the same procedure, but the cecum was neither ligated nor punctured. MaR1 (100 ng/mice) was intraperitoneally administered 1 h later after surgery. The experimental schedule is shown in Figure 1(b).

### 2.3. Survival Analysis

The mice which had undergone CLP surgery were randomly administered MaR1 (100 ng) or saline in the sham group (intraperitoneal injection; *n* = 12/per group). The survival rate of mice was monitored for 8 days after CLP surgery and recorded every day.

### 2.4. Pathological and Biological Studies

The lung and liver samples were obtained immediately after exsanguination (24 h after CLP) then embedded in paraffin and stained with haematoxylin and eosin (H&E) for light microscope analysis. Organ injury scores were performed by two independent investigators blinded to the research assignment following the applicable criteria [16]. Whole blood samples from mice were obtained by cardiac puncture 24 h after CLP surgery and then centrifuged at 3000 g for 10 min to collect serum. The serum levels of TNF–*α*, IL-6, alanine aminotransferase (ALT), and aspartate aminotransferase (AST) were, respectively, determined using ELISA kits according to the manufacturer's protocols.

### 2.5. Transmission Electron Microscopy

For electron microscopy experiments, the lung tissues were harvested 24 h after CLP and the effect of MaR1 was assessed by transmission electron microscopy analysis. Mice were euthanized and then perfused with cold PBS, followed by 2% glutaraldehyde in 0.1 M phosphate buffer (pH 7.4), and processed for transmission electron microscopy (TEM), as described previously [17]. Images were acquired digitally from a randomly selected pool of 10 fields under each condition.

### 2.6. ^1^H NMR Sample Collection and Extraction

Lung and liver samples were collected, frozen in liquid nitrogen rapidly, and stored at −80°C until use. The extraction was performed as previously described [18]. Prior to NMR analysis, the tissue sample was weighed into an Eppendorf tube, mixed with ice-cold methanol (4 mL/g) and distilled water (0.85 mL/g), and homogenized by a handheld homogenizer. Then, ice-cold chloroform (2 mL/g) and distilled water (2 mL/g) were added into the mixture, vigorously vortex-mixed for 10 s, and centrifuged at 1000 g for 15 min at 4°C. The supernatant was extracted carefully into a new tube and lyophilized for 48 h. The dried extracts were resuspended in 500 *μ*L D_2_O containing sodium trimethylsilyl propionate-d_4_ (TSP, 0.25 mM) and then transferred to a 5 mm NMR tube for NMR analysis. In addition, 200 *μ*L of serum sample was thawed and diluted with 250 *μ*L of phosphate buffer and 50 *μ*L of D_2_O. The diluted serum was mixed and centrifuged at 12,000 g at 4°C for 15 min. After that, 500 *μ*L of supernatant was transferred into a NMR tube for analysis.

### 2.7. ^1^H NMR Spectrometry

The ^1^H NMR spectra were measured by using a Bruker Avance III 600 spectrometer equipped with a triple resonance probe (Bruker BioSpin, Rheinstetten, Germany) at 128 K. A standard single-pulse sequence (ZGPR) with water signal presaturation was employed to acquire NMR spectra of tissue extracts. For serum sample, the Carr-Purcell-Meiboom-Gill (CPMG) pulse sequence with a fixed receiver-gain value was performed in order to minimize broad NMR peaks from proteins and lipids. The main acquisition parameters were set as follows: spectral width = 12,000 Hz, data points = 256 K, relaxation delay = 4 s, and acquisition time = 2.66 s per scan.

The ^1^H NMR spectra were corrected manually for phase and baseline using TopSpin 3.0 software (Bruker BioSpin, Rheinstetten, Germany). The spectra of serum samples were referenced to the methyl peak of lactate at 1.33 ppm, while the spectra of tissue samples were referenced to the TSP peak at 0 ppm. The “icoshift” procedure was used to align NMR spectra in MATLAB software (R2012a, The MathWorks Inc., Natick, MA, USA) [19]. The spectral regions from 0.5 to 10.0 ppm excluding the residual water signals (from 4.7 to 5.2 ppm) were subdivided and integrated to binning data with a size of 0.01 ppm.

### 2.8. Multivariate and Statistical Analysis

In this study, all mice were randomly assigned to experimental procedures including housing and feeding, animal grouping, CLP surgery, and MaR1 treatment. Principal component analysis (PCA) was used to obtain an overview of the metabolic pattern of animal models using the SIMCA software (v. 12.0, Umetrics, Umeå, Sweden). Prior to PCA, NMR data were Pareto-scaled for improving data comparability. All data were analyzed by one-way analysis of variance (ANOVA) followed by Fisher's LSD test using SPSS software (version 13.0, SPSS). Survival of the two subgroups was estimated by Kaplan–Meier survival curves and compared by the log-rank test. In this study, a statistically significant difference was considered when *P* value <0.05. Heat map and metabolic pathway analyses were conducted by using MetaboAnalyst 3.0 [20]. The result of pathway analysis was presented according to *P* values from the pathway enrichment analysis and pathway impact values from the pathway topology analysis, and the metabolic pathway with high values of these two parameters, namely, located in the top-right region, was identified as the most important pathway.

## 3. Results

### 3.1. MaR1 Improved Survival Rate and Alleviated Lung and Liver Injury in CLP-Induced Septic Mice

**Figure 2:**
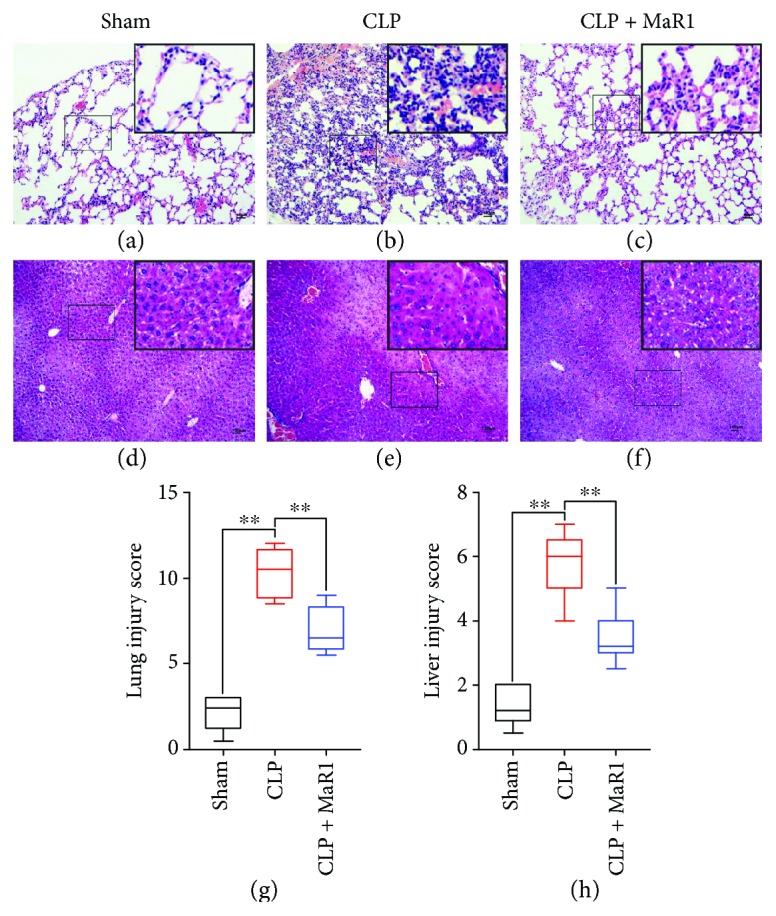
MaR1 attenuated lung and liver injury in CLP-induced septic mice. The lung and liver tissues were obtained immediately after exsanguination (24 h after CLP), and the effect of MaR1 was assessed histologically in H&E-stained sections (original magnification ×100). Inset shows 40x magnification of the slides. (a-c) Lung tissues, (d-f) liver tissues, (g) lung injury scores (*n* = 5 per group), and (h) liver injury scores (*n* = 5 per group). Data are presented as mean ± SD. ^∗^*P* < 0.05 and ^∗∗^*P* < 0.01, *n* = 5 per group.

As shown in Figure 1(c), MaR1 significantly increased the survival rate compared with the septic group (58.33% versus 16.67%; *P* < 0.05). Pathologic examination revealed that mice in the sham group displayed normal tissue histology (Figures 2(a) and 2(d)). After CLP, the lung was characterized by neutrophil infiltration, haemorrhage, alveolar disarray, and hyaline membrane (Figure 2(b)), while the liver showed increased parenchymal inflammatory and degenerative changes (Figure 2(e)). However, MaR1 administration obviously attenuated lung and liver injury (Figures 2(c) and 2(f)), which is further confirmed by organ injury scores (Figures 2(g) and 2(h)).

### 3.2. MaR1 Attenuated TNF-*α*, IL-6, and ALT/AST Levels in CLP-Induced Septic Mice

**Figure 3:**
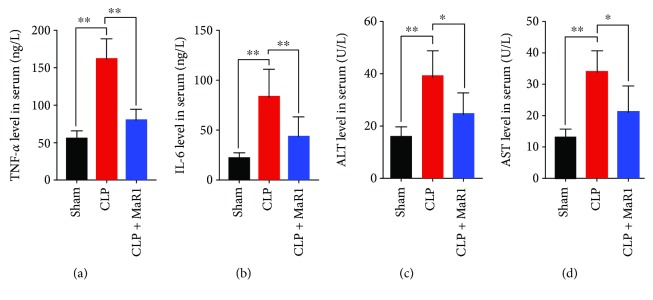
MaR1 reduced TNF-*α*, IL-6, and ALT/AST levels in CLP-induced septic mice. Blood samples were collected after CLP surgery at 24 h time point and then centrifuged at 3000 g for 10 min to collect serum. (a, b) The serum levels of TNF–*α* and IL-6 were, respectively, determined using ELISA kits. (c, d) Then, the levels of alanine aminotransferase (ALT) and aspartate aminotransferase (AST) in blood were determined by biochemistry assay kits. Data are presented as mean ± SD. ^∗^*P* < 0.05 and ^∗∗^*P* < 0.01, *n* = 5 per group.

To assess the effect of MaR1 on inflammatory response in CLP-induced septic mice, the concentrations of proinflammatory cytokines in serum were detected at 24 h after CLP. The levels of TNF-*α* and IL-6 were significantly increased in serum of CLP-induced septic mice compared with the sham group (Figures 3(a) and 3(b)). However, after MaR1 treatment, TNF-*α* and IL-6 levels were markedly reduced in serum of CLP-induced septic mice (Figures 3(a) and 3(b)). In addition, we found that the levels of ALT and AST were increased significantly in CLP-induced septic mice as compared with the sham group (*P* < 0.01), while their levels were notably downregulated after MaR1 treatment (Figures 3(c) and 3(d)).

### 3.3. MaR1 Mitigated Mitochondrial Damage in CLP-Induced Septic Mice

**Figure 4:**
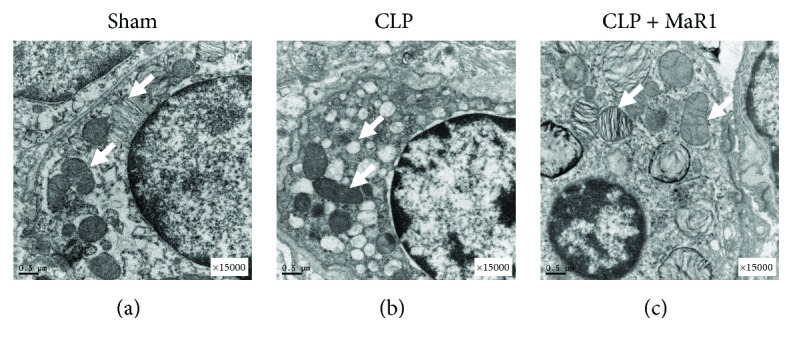
MaR1 mitigated mitochondrial damage in CLP-induced septic mice. For electron microscopy experiments, the lung tissues were harvested 24 h after CLP and the effect of MaR1 was assessed by transmission electron microscopy analysis (original magnification ×15,000). The mitochondrial ultrastructure changes in three experimental groups were indicated by the white arrows.

As shown in Figures 4(a)–4(c), the mitochondria in lung tissues from the CLP group were swollen with disrupted or disintegrated cristae, and the osmiophilic lamellar bodies had fused or disappeared. This mitochondrial damage was mitigated in the CLP + MaR1 group.

### 3.4. MaR1 Affected Metabolic Features of Serum and Lung but Not Liver in CLP-Induced Septic Mice

**Figure 5:**
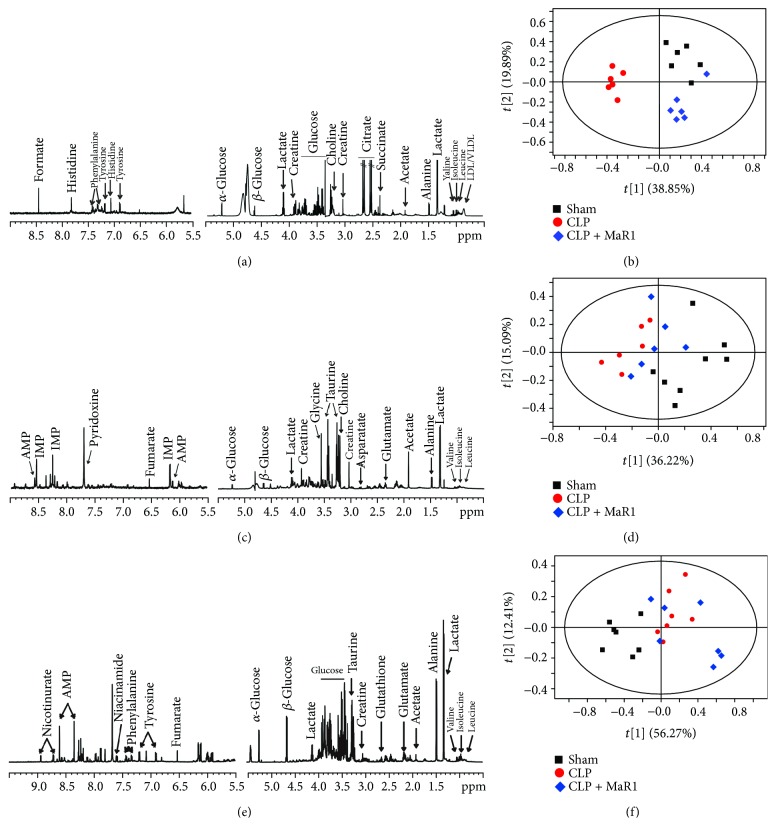
^1^H-NMR-based metabolic profiles patterns in septic mouse treatment with MaR1. Representative ^1^H NMR spectra of serum (a), lung (c), and liver (e) samples obtained from the mice, respectively. The PCA score plot based on the 1H NMR spectra of serum (b), lung (d), and liver (f) samples obtained from mice of three experimental groups.

Typical ^1^H NMR spectra in serum, liver, and lung samples are illustrated in Figures 5(a), 5(c), and 5(e). A series of metabolites were identified, involving energy metabolisms (acetate, creatine, glucose, lactate and pyruvate, and formate), lipid metabolism (LDL/VLDL), amino acid metabolism (alanine, glutamine, glycine, histidine, isoleucine, leucine, phenylalanine, tyrosine, and valine), and ketone body metabolism (acetone, acetoacetate, and 3-hydroxybutyrate).

In this study, PCA was applied to examine changes in metabolic patterns among sham, CLP, and MaR1 groups. PCA can clearly distinguish among these three groups based on serum metabolome (Figure 5(b)). In addition, Figure 5(d) indicates that lung metabolic patterns were also different among these three groups. For liver metabolome, the metabolic pattern in the sham group was clearly separated from the other two groups, while no obvious separation was obtained between CLP and CLP + MaR1 groups (Figure 5(f)).

**Figure 6:**
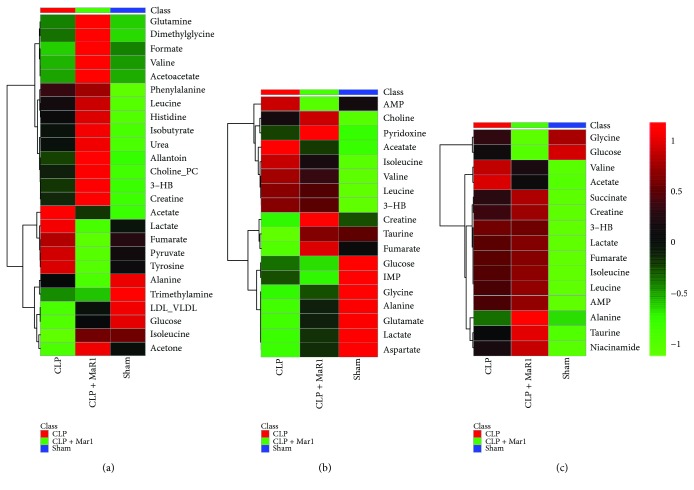
Heat map visualization of the identified differential metabolites in septic mouse treatment with MaR1. Heat maps of metabolites in serum (a), lungs (b), and liver (c) of septic mouse treatment with MaR1.

Furthermore, metabolites were quantified and illustrated in a heat map (Figure 6). As can be seen from Figure 6(a), the CLP + MaR1 group had higher levels of acetate, lactate, fumarate, pyruvate, and tyrosine than the sham group, but their levels were obviously reduced after MaR1 treatment. Additionally, relative to the sham group, the levels of LDL/VLDL, glucose, isoleucine, and acetone levels were reduced in serum of CLP-induced septic mice. Of note, MaR1 treatment markedly increased these metabolites in serum (Figure 6(a)). However, there were no recovery trends for other serum metabolites shown in Figure 6(a).

In the lung, we also identified several metabolites that show a recovery trend after MaR1 treatment; for example, acetate and isoleucine levels were apparently increased in the CLP group relative to the sham group but decreased in the CLP + MaR1 group (Figure 6(b)). A decrease in lung taurine caused by CLP was recovered to the normal level after MaR1 treatment. In addition, a slight recovery trend was also found for glycine, alanine, glutamate, lactate, and aspartate in lung, as shown in Figure 6(b).

Most metabolites in the liver did not show a recovery trend, but increased levels of valine and acetate in the CLP group relative to the sham group were mildly reduced after MaR1 administration (Figure 6(c)).

### 3.5. Metabolic Pathway Changes in CLP-Induced Septic Mice after MaR1 Treatment

**Figure 7:**
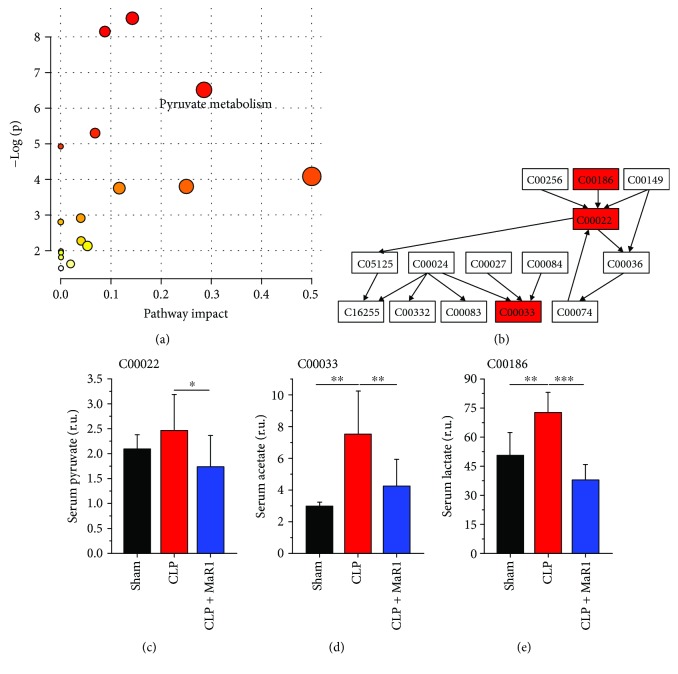
Metabolic pathway analysis in serum of septic mouse treatment with MaR1. Pathway analysis carried out by the pathway topology analysis among three groups (a) and the pathway flowchart of pyruvate metabolism (b). The key metabolites are shown in (c)~(e). ^∗^*P* < 0.05, ^∗∗^*P* < 0.01, and ^∗∗∗^*P* < 0.001, *n* = 6 per group.

Furthermore, metabolic pathway analysis was performed on the basis of metabolites that show a recovery trend after MaR1 treatment.  Figure 7(a) illustrates an overview of pathway analysis using serum metabolites according to *P* values from the pathway enrichment analysis and pathway impact values from the pathway topology analysis. Pyruvate metabolism was identified as a key metabolic pathway affected by MaR1 treatment due to its relatively high pathway impact value and statistically significance (Figure 7(a)). In the present study, we detected three metabolites, pyruvate, acetate, and lactate, in pyruvate metabolism via NMR-based metabolomics (Figure 7(b)). Results show that CLP-induced septic mice had higher levels of pyruvate (Figure 7(c)), acetate (Figure 7(d)), and lactate (Figure 7(e)) in serum as compared with normal control mice, but their levels were significantly reduced after MaR1 treatment.

**Figure 8:**
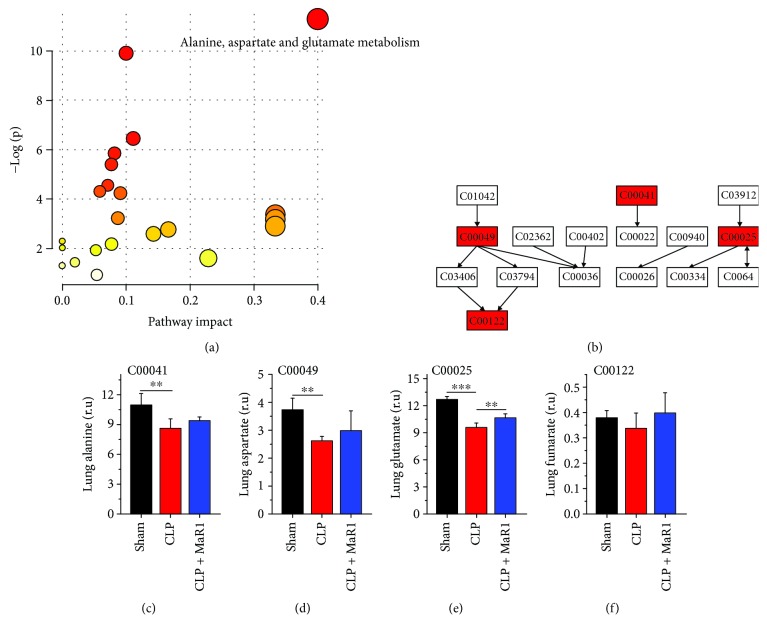
Metabolic pathway analysis in lung of septic mouse treatment with MaR1. Pathway analysis carried out by the pathway topology analysis among three groups (a) and the pathway flowchart of alanine, aspartate, and glutamate metabolism (b). The key metabolites are shown in (c)~(f). ^∗∗^*P* < 0.01 and ^∗∗∗^*P* < 0.001, *n* = 6 per group.

In lung tissue, we identified alanine, aspartate, and glutamate metabolism as a key metabolic pathway (Figures 8(a) and 8(b)). Compared with the sham group, the levels of alanine (Figure 8(c), *P* < 0.01), aspartate (Figure 8(d), *P* < 0.01), glutamate (Figure 8(e), *P* < 0.001), and fumarate (Figure 8(f), *P* > 0.05) were decreased in lung tissue of the CLP group; however, their levels were marginally increased after MaR1 treatment.

## 4. Discussion

Maresin1 (MaR1) is a new family of special proresolving mediators (SPMs) that are derived from endogenous DHA. SPMs have been shown to exert potent protective effects in humans' biology and health [21, 22]. Our results show that the treatment of MaR1 markedly increased the survival rate and alleviated lung and liver injury in CLP-induced septic mice. In addition, our data demonstrate that MaR1 treatment significantly reduced the levels of TNF-*α* and IL-6 in plasma, which may influence the patients' outcome in critical illness [23]. Korner et al. reported that Omega-3 fatty acids reduced inflammation and improved sepsis survival in mice by stimulating the endogenous production of MaR1 [24]. Moreover, the organ-protective effect of MaR1 was also observed in murine sepsis by several researchers [6, 14, 25].

Although MaR1 has demonstrated a potential protective effect on sepsis, its clinical application is still in its infancy [21]. Exploring the metabolic mechanisms of MaR1 will promote its clinical development. In our previous study, for example, we used an NMR-based metabolomics analysis to examine the metabolic responses of the basic fibroblast growth factor in streptozotocin-induced diabetic rats and provide several possible mechanisms of the glucose/lipid-lowering effect [18]. Herein, we investigated metabolic changes in CLP-induced septic mice after MaR1 treatment using NMR-based metabolomics and found two main metabolic pathways recovered by MaR1, including pyruvate metabolism and alanine, aspartate, and glutamate metabolism.

### 4.1. MaR1 Recovered Pyruvate Metabolism in CLP-Induced Septic Mice

Pyruvate metabolism has been closely associated with human disease by maintaining energy metabolism homeostasis [26]. Pyruvate is formed from glucose through glycolysis and then oxidized to CO_2_ and H_2_O via the tricarboxylic acid (TCA) cycle or transformed to lactate by anaerobic glycolysis. In the present study, our results reveal that MaR1 can effectively recover abnormal increases in serum pyruvate and lactate levels in CLP-induced septic mice, suggesting a recovery of pyruvate metabolism after MaR1 treatment. Of note, lactate is an established indicator in tissue hypoxia and mitochondrial dysfunction in critical illness [27, 28]. Recent findings in septic patients have reported an association between increased serum lactate level with organ dysfunction and outcomes [29, 30]. Our previous study also found that MaR1 remarkably decreased the serum level of lactate in septic mice [14]. Therefore, MaR1 may improve lactate clearance in sepsis. Moreover, these findings supported MaR1-induced alleviation of the mitochondrial damage from the metabolic perspective. Acetate, as a contributor of the TCA cycle, is indirectly related to pyruvate metabolism. Stringer et al. [11] detected a slight increase in plasma acetate level in patients with sepsis-induced acute lung injury relative to healthy controls. In our study, as compared with normal control mice, serum acetate level was significantly increased in CLP-induced septic mice. Yet, it should be noted that this increase was effectively recovered after MaR1 treatment. Bakalov et al. [31] reported that a reduced acetate level may be a metabolic feature in a *Drosophila melanogaster* model of surviving sepsis. Taken together, the recovery of pyruvate metabolism induced by MaR1 treatment could be contributed to its potential therapeutic effect in CLP-induced septic mice.

### 4.2. MaR1 Recovered Alanine, Aspartate, and Glutamate Metabolism in CLP-Induced Septic Mice

Metabolomics results also reveal that MaR1 treatment recovered abnormal metabolism of alanine, aspartate, and glutamate in CLP-induced septic mice. Glutamate plays a vital role in the biosynthesis of proteins in living beings. The reduction in glutamate level in plasma has been linked to poor outcomes in septic shock patients [32]. In the present study, we found that the glutamate level in the lung was significantly decreased in the CLP group. Reduced lung glutamate production can result in a decrease in systemic glutamate availability in hyperdynamic sepsis [33]. Alteration in glutamate metabolism may also influence Th17 cell differentiation by affecting methylation of Foxp3 in autoimmune disease [34]. MaR1 could augment the generation of Tregs and regulated cytokine production from ILC2s in the allergic lung inflammatory model [35]. Thus, MaR1 significantly increased the level of glutamate in the lung, indicating that MaR1 may affect T cell responses via maintaining glutamate metabolism homeostasis in sepsis. However, their causal relationships need to be further explored.

Alanine has a close association with multiple metabolic pathways such as glycolysis, gluconeogenesis, and the TCA cycle. In this study, the alanine level in lung tissue was significantly reduced in CLP-induced septic mice as compared with normal control mice. This finding may be due to a significant increase in ALT level in CLP-induced septic mice, since ALT can catalyse the transfer of an amino group from alanine to *α*-ketoglutarate and form pyruvate and glutamate. However, Izquierdo-Garcia et al. [13] found an increased level of alanine in lung tissue of septic rats induced by cecal ligation and puncture. We speculate that this difference could be attributed to different sepsis modelling procedures. Additionally, we found that MaR1 treatment slightly increased the level of alanine in the lung of CLP-induced septic mice.

Aspartate is synthesized from an intermediate of the TCA cycle, oxaloacetate, by transamination. Our results show that MaR1 treatment mildly recovered the CLP-induced reduction in lung aspartate level. The same trend was also obtained in the level of lung fumarate, which is an important intermediate of the TCA cycle. Therefore, MaR1 could maintain energy metabolism homeostasis in the lung of CLP-induced septic mice. Consistent with this finding, we also found that MaR1 ameliorated the CLP-induced ultrastructural damage of mitochondria in lung tissue by transmission electron microscopy analysis.

### 4.3. MaR1 Improved Taurine Level in Lung of CLP-Induced Septic Mice

Oxidative stress has been implicated in the pathogenesis of sepsis [36, 37]. Taurine can enhance the ability of the antioxidant defence system by protecting antioxidant enzymes [38]. In this study, a significant decrease in taurine level was detected in the lung of CLP-induced septic mice as compared with normal control mice, suggesting a damaged antioxidant defence system in septic mice. However, of note, we found that MaR1 treatment obviously increased the lung taurine level. The inhibitory effect of MaR1 on oxidative stress was also reported in lung I/R injury and carbon tetrachloride-induced hepatic injury [39, 40]. Thus, we speculate that the potential therapeutic effect of MaR1 on CLP-induced sepsis may be linked with the recovery of taurine-mediated defence against oxidative stress.

## 5. Conclusions

The present study suggested that MaR1 treatment improved the survival rate of CLP-induced septic mice in multiple ways, including the recovery of lung and liver injuries, the reduction of inflammatory cytokines, and the attenuation of mitochondrial damage. Furthermore, using a metabolomics analysis, we also found that MaR1 could alleviate metabolic disorders in CLP-induced septic mice, mainly involving pyruvate metabolism, alanine, aspartate, and glutamate metabolism, as well as lung taurine level. Yet, it is worth noting that MaR1 significantly regulated the metabolic changes in serum and lung but not liver in septic mice. The potential explanation underlying this phenomenon could be due to the time effect of MaR1 treatment on sepsis. Therefore, the impacts of MaR1 on hepatic metabolic response might occur for an extended period. Our findings reveal a novel metabolic mechanism for the anti-inflammatory and proresolving actions of MaR1 in sepsis. MaR1 may provide new opportunities to design “metabolic targeted” therapies with high accuracy in treating sepsis. However, several limitations or future works should be considered: on the one hand, based on the present preliminary study, cause-effect associations among MaR1, metabolism, and sepsis cannot be proved. Therefore, cellular experiments or other animal models are recommended to discover more evidence on the therapeutic mechanisms of MaR1 in sepsis. On the other hand, metabolomics needs to be coupled with other omics techniques (e.g., genomics and proteomics) for a better understanding of the effect of MaR1 on metabolic regulation and sepsis treatment. We believe that a deeper insight into the MaR1 therapeutic mechanisms will promote its clinical development and application.

## Data Availability

The data used to support the findings of this study are available from the corresponding author upon request.

## References

[1] Singer M., Deutschman C. S., Seymour C. W. (2016). The Third International Consensus Definitions for Sepsis and Septic Shock (Sepsis-3). *JAMA*.

[2] Hotchkiss R. S., Moldawer L. L., Opal S. M., Reinhart K., Turnbull I. R., Vincent J. L. (2016). Sepsis and septic shock. *Nature Reviews Disease Primers*.

[3] Serhan C. N., Yang R., Martinod K. (2009). Maresins: novel macrophage mediators with potent antiinflammatory and proresolving actions. *The Journal of Experimental Medicine*.

[4] Colas R. A., Dalli J., Chiang N. (2016). Identification and actions of the Maresin 1 metabolome in infectious inflammation. *Journal of Immunology*.

[5] Serhan C. N., Dalli J., Colas R. A., Winkler J. W., Chiang N. (2015). Protectins and maresins: new pro-resolving families of mediators in acute inflammation and resolution bioactive metabolome. *Biochimica et Biophysica Acta*.

[6] Li R., Wang Y., Ma Z. (2016). Maresin 1 mitigates inflammatory response and protects mice from sepsis. *Mediators of Inflammation*.

[7] Zhang J. L., Zhuo X. J., Lin J. (2017). Maresin1 stimulates alveolar fluid clearance through the alveolar epithelial sodium channel Na,K-ATPase via the ALX/PI3K/Nedd4-2 pathway. *Laboratory Investigation*.

[8] Wishart D. S. (2016). Emerging applications of metabolomics in drug discovery and precision medicine. *Nature Reviews Drug Discovery*.

[9] Singh S., Chatterji T., Sen M. (2016). Serum procalcitonin levels in combination with ^1^H NMR spectroscopy: a rapid indicator for differentiation of urosepsis. *Clinica Chimica Acta*.

[10] Stringer K. A., McKay R. T., Karnovsky A., Quemerais B., Lacy P. (2016). Metabolomics and its application to acute lung diseases. *Frontiers in Immunology*.

[11] Stringer K. A., Serkova N. J., Karnovsky A., Guire K., Paine R., Standiford T. J. (2011). Metabolic consequences of sepsis-induced acute lung injury revealed by plasma ^1^H-nuclear magnetic resonance quantitative metabolomics and computational analysis. *American Journal of Physiology Lung Cellular and Molecular Physiology*.

[12] Lin Z. Y., Xu P. B., Yan S. K. (2009). A metabonomic approach to early prognostic evaluation of experimental sepsis by (1)H NMR and pattern recognition. *NMR in Biomedicine*.

[13] Izquierdo-Garcia J. L., Nin N., Ruiz-Cabello J. (2011). A metabolomic approach for diagnosis of experimental sepsis. *Intensive Care Medicine*.

[14] Gu J., Luo L., Wang Q. (2018). Maresin 1 attenuates mitochondrial dysfunction through the ALX/cAMP/ROS pathway in the cecal ligation and puncture mouse model and sepsis patients. *Laboratory Investigation*.

[15] Rittirsch D., Huber-Lang M. S., Flierl M. A., Ward P. A. (2009). Immunodesign of experimental sepsis by cecal ligation and puncture. *Nature Protocols*.

[16] Wang Q., Zheng X., Cheng Y. (2014). Resolvin D1 stimulates alveolar fluid clearance through alveolar epithelial sodium channel, Na,K-ATPase via ALX/cAMP/PI3K pathway in lipopolysaccharide-induced acute lung injury. *Journal of Immunology*.

[17] Zhuo X. J., Hao Y., Cao F. (2018). Protectin DX increases alveolar fluid clearance in rats with lipopolysaccharide-induced acute lung injury. *Experimental & Molecular Medicine*.

[18] Lin X., Zhao L., Tang S. (2016). Metabolic effects of basic fibroblast growth factor in streptozotocin-induced diabetic rats: a ^1^H NMR-based metabolomics investigation. *Scientific Reports*.

[19] Savorani F., Tomasi G., Engelsen S. B. (2010). icoshift: a versatile tool for the rapid alignment of 1D NMR spectra. *Journal of Magnetic Resonance*.

[20] Xia J., Sinelnikov I. V., Han B., Wishart D. S. (2015). MetaboAnalyst 3.0—making metabolomics more meaningful. *Nucleic Acids Research*.

[21] Serhan C. N. (2017). Discovery of specialized pro-resolving mediators marks the dawn of resolution physiology and pharmacology. *Molecular Aspects of Medicine*.

[22] Wu S. H., Chen X. Q., Liu B., Wu H. J., Dong L. (2013). Efficacy and safety of 15(R/S)-methyl-lipoxin A_4_ in topical treatment of infantile eczema. *The British Journal of Dermatology*.

[23] Casey L. C., Balk R. A., Bone R. C. (1993). Plasma cytokine and endotoxin levels correlate with survival in patients with the sepsis syndrome. *Annals of Internal Medicine*.

[24] Korner A., Schlegel M., Theurer J. (2018). Resolution of inflammation and sepsis survival are improved by dietary Ω-3 fatty acids. *Cell Death & Differentiation*.

[25] Gong J., Liu H., Wu J. (2015). Maresin 1 prevents lipopolysaccharide-induced neutrophil survival and accelerates resolution of acute lung injury. *Shock*.

[26] de Meirleir L., Garcia-Cazorla A., Brivet M., Fernandes J., Saudubray J. M., Baumgartner M., Walter J. (2016). Disorders of pyruvate metabolism and the tricarboxylic acid cycle. *Inborn Metabolic Diseases*.

[27] Shapiro N. I., Howell M. D., Talmor D. (2005). Serum lactate as a predictor of mortality in emergency department patients with infection. *Annals of Emergency Medicine*.

[28] Zhang Z., Xu X. (2014). Lactate clearance is a useful biomarker for the prediction of all-cause mortality in critically ill patients: a systematic review and meta-analysis. *Critical Care Medicine*.

[29] Hayashida K., Suzuki M., Yonemoto N. (2017). Early lactate clearance is associated with improved outcomes in patients with postcardiac arrest syndrome: a prospective, multicenter observational study (SOS-KANTO 2012 study). *Critical Care Medicine*.

[30] Nguyen H. B., Rivers E. P., Knoblich B. P. (2004). Early lactate clearance is associated with improved outcome in severe sepsis and septic shock. *Critical Care Medicine*.

[31] Bakalov V., Amathieu R., Triba M. N. (2016). Metabolomics with nuclear magnetic resonance spectroscopy in a *Drosophila melanogaster* model of surviving sepsis. *Metabolites*.

[32] Poeze M., Luiking Y. C., Breedveld P., Manders S., Deutz N. E. (2008). Decreased plasma glutamate in early phases of septic shock with acute liver dysfunction is an independent predictor of survival. *Clinical Nutrition*.

[33] Ten Have G. A., Engelen M. P., Wolfe R. R., Deutz N. E. (2012). Reduced lung glutamate (GLU) production is the cause of decreased systemic glutamate availability in hyperdynamic sepsis. *The FASEB Journal*.

[34] Xu T., Stewart K. M., Wang X. (2017). Metabolic control of T_H_17 and induced T_reg_ cell balance by an epigenetic mechanism. *Nature*.

[35] Chiurchiu V., Leuti A., Dalli J. (2016). Proresolving lipid mediators resolvin D1, resolvin D2, and maresin 1 are critical in modulating T cell responses. *Science Translational Medicine*.

[36] Prauchner C. A. (2017). Oxidative stress in sepsis: pathophysiological implications justifying antioxidant co-therapy. *Burns*.

[37] Molina V., von Dessauer B., Rodrigo R., Carvajal C. (2017). Oxidative stress biomarkers in pediatric sepsis: a prospective observational pilot study. *Redox Report*.

[38] Zhang H., Hu C. A., Kovacs-Nolan J., Mine Y. (2015). Bioactive dietary peptides and amino acids in inflammatory bowel disease. *Amino Acids*.

[39] Sun Q., Wu Y., Zhao F., Wang J. (2017). Maresin 1 ameliorates lung ischemia/reperfusion injury by suppressing oxidative stress via activation of the Nrf-2-mediated HO-1 signaling pathway. *Oxidative Medicine and Cellular Longevity*.

[40] Li R., Wang Y., Zhao E. (2016). Maresin 1, a proresolving lipid mediator, mitigates carbon tetrachloride-induced liver injury in mice. *Oxidative Medicine and Cellular Longevity*.

